# Pictilisib-Induced Resistance Is Mediated through FOXO1-Dependent Activation of Receptor Tyrosine Kinases in Mucinous Colorectal Adenocarcinoma Cells

**DOI:** 10.3390/ijms241512331

**Published:** 2023-08-02

**Authors:** Murali R. Kuracha, Venkatesh Govindarajan, Brian W. Loggie, Martin Tobi, Benita L. McVicker

**Affiliations:** 1Department of Internal Medicine, University of Nebraska Medicine, Omaha, NE 68198, USA; 2Department of Medical Education, Creighton University School of Medicine, Omaha, NE 68178, USA; 3Department of Surgery, Creighton University School of Medicine, Omaha, NE 68124, USA; 4Research and Development Service, Detroit VAMC, Detroit, MI 48201, USA; 5Research Service, Nebraska-Western Iowa Health Care System, Omaha, NE 68105, USA

**Keywords:** FOXO, pictilisib, mucinous colorectal adenocarcinomas

## Abstract

The phosphatidylinositol (PI3K)/AKT/mTOR axis represents an important therapeutic target to treat human cancers. A well-described downstream target of the PI3K pathway is the forkhead box O (FOXO) transcription factor family. FOXOs have been implicated in many cellular responses, including drug-induced resistance in cancer cells. However, FOXO-dependent acute phase resistance mediated by pictilisib, a potent small molecule PI3K inhibitor (PI3Ki), has not been studied. Here, we report that pictilisib-induced adaptive resistance is regulated by the FOXO-dependent rebound activity of receptor tyrosine kinases (RTKs) in mucinous colorectal adenocarcinoma (MCA) cells. The resistance mediated by PI3K inhibition involves the nuclear localization of FOXO and the altered expression of RTKs, including *ErbB2*, *ErbB3*, *EphA7*, *EphA10*, *IR*, and *IGF-R1* in MCA cells. Further, in the presence of FOXO siRNA, the pictilisib-induced feedback activation of RTK regulators (pERK and pAKT) was altered in MCA cells. Interestingly, the combinational treatment of pictilisib (Pi3Ki) and FOXO1i (AS1842856) synergistically reduced MCA cell viability and increased apoptosis. These results demonstrate that pictilisib used as a single agent induces acute resistance, partly through FOXO1 inhibition. Therefore, overcoming PI3Ki single-agent adaptive resistance by rational design of FOXO1 and PI3K inhibitor combinations could significantly enhance the therapeutic efficacy of PI3K-targeting drugs in MCA cells.

## 1. Introduction

Phosphatidylinositol 3-kinase (PI3K)-targeting drugs are creating great interest as therapeutics for human cancers [1]. PI3Ks belong to the lipid kinase family that is divided into three classes. Class I is further subdivided into classes IA and IB, with class IA PI3Ks implicated in human cancers [2]. Class IA PI3Ks are heterodimeric, comprised of a regulatory subunit (p85α, p55α, p50α, p85β, and p55γ) and a catalytic subunit (p110α, p110β, and p110γ) encoded by the gene PI3KCA [3]. Commonly, PI3KCA gene mutations occur in the kinase domain (e.g., H1047R) or in the regulatory domain (e.g., E542K and E545K). These mutations diminish inhibitory interactions leading to constitutive activation of PI3K and downstream effectors (e.g., AKT, mTOR, and S6 kinase) [4]. The hyperactivation of these signaling nodes regulates the PI3K/AKT axis towards the growth and survival of the cancer cell [2]. However, several small molecule inhibitors that target the key nodes of the PI3K pathway have been developed and are currently in various preclinical and clinical trials [5]. Effective PI3K-inhibiting drugs are especially desired for hard-to-treat aggressive tumors. Mucinous colorectal adenocarcinomas (MCA) tend to be very aggressive and comprise a significant portion (10–20%) of colorectal cancers (CRC). MCAs are a highly disseminated heterogeneous group of tumors with a propensity to metastasize to the liver and peritoneal cavity [6,7,8,9,10,11]. Clinically, higher mutational rates for *KRAS*, *BRAF*, and PI3KCA have been reported in MCA rather than non-mucinous CRCs [12]. Pictilisib is a small molecule inhibitor of PI3K that has been used in chemotherapeutic regimens for a variety of human cancers, including MCA [13,14]. However, the effectiveness of pictilisib as a single-agent therapy is limited due to the potential development of drug-induced resistance.

Forkhead (FOXO) family transcription factors, FOXO1, FOXO3a, and FOXO4, are direct downstream targets of the PI3K/AKT pathway [15,16]. Importantly, FOXO proteins are involved in several post-translational modifications, including cell cycle inhibition [17], apoptosis [18,19], defense against oxidative stress, and DNA repair [20,21]. Moreover, FOXOs can act as critical modulators in cellular processes, including drug-induced resistance in many cell types [18,22]. In particular, FOXO1 is known to modulate paclitaxel-mediated cytotoxic resistance in ovarian cancers [23,24]. Such drug-induced resistance in cancer cells could be a result of FOXO1 nuclear migration and subsequent transcriptional activity. Currently, there is limited information concerning mechanisms and transcriptional changes mediated by drugs that induce resistance in cancer cells.

In the present study, we examined the consequence of FOXO1 nuclear localization in mucinous colorectal cancer cell lines associated with PI3K inhibition and pictilisib-induced resistance.

## 2. Results

### 2.1. Nuclear Localization of FOXO Transcription Factors after Pi3K Inhibition in MCA Cells

**Figure 1 ijms-24-12331-f001:**
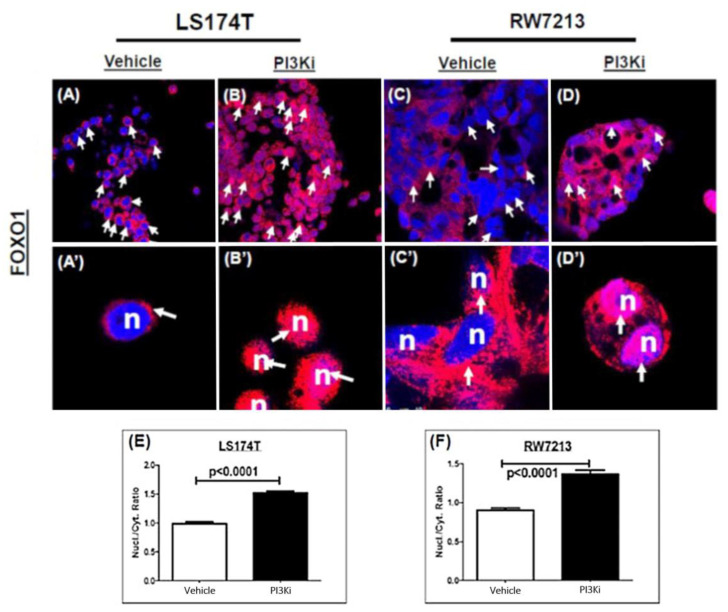
Nuclear enrichment of FOXOs in PI3Ki-treated MCA cells. (**A**,**A’**,**C,C’**): DMSO vehicle-treated LS174T and RW7213 cells expressed FOXO1 after 96 h. (**B**,**B’**,**D**,**D’**): LS174T and RW7213 cells exhibit FOXO1 staining in the nuclear compartment after 96 h of pictilisib treatment. Nuclei were assigned with pseudo-colored blue and FOXO1 with red. Nuclear enrichment of FOXO1 was seen at 96 h in both MCA cell lines (nuclei appear pink due to the red–blue overlap (white arrows). Cytoplasmic FOXO1 peri nuclear red-stained in (**A’**,**C’**) (white arrows). Scale bar: (**A**–**D**): 25 μm; (**A’**,**B’**,**D’**): 7.5 μm; (**C’**):10 μm. Statistical significance of nuclear FOXO1 staining intensity was measured in both MCA cell lines using a two-tail *t*-test (**E**,**F**): LS174T (vehicle, *n* = 230 cells; PI3Ki, *n* = 249 cells), RW7213 (vehicle = DMSO control, *n* = 236 cells; PI3Ki, *n* = 114 cells). *p*-value significance *p* < 0.001; error bars: standard error mean values were plotted.

FOXO protein localization in response to PI3K inhibition in mucinous colorectal cancer cells (LS174T and RW7213) was examined using immunocytochemistry. Interestingly, FOXO1 was strongly stained in the nuclear compartment in MCA cells treated with pictilisib for 96 h compared to DMSO-treated control cells. In both MCA cell lines, the staining intensity of FOXO1 was significantly elevated in response to pictilisib single-agent treatment (Figure 1A–D’). Quantification of nuclear stain intensity showed a significant increase in the number of FOXO1 nuclear-stained positive cells in pictilisib-treated compared to DMSO-treated LS174T and RW7213 cells (Figure 1E,F). It was also determined that the FOXO3 protein was nuclear-localized following PI3K inhibition in the MCA cells.

### 2.2. Characterization of FOXO Nuclear Migration following Pictilisib Treatment in LS174T Cells

**Figure 2 ijms-24-12331-f002:**
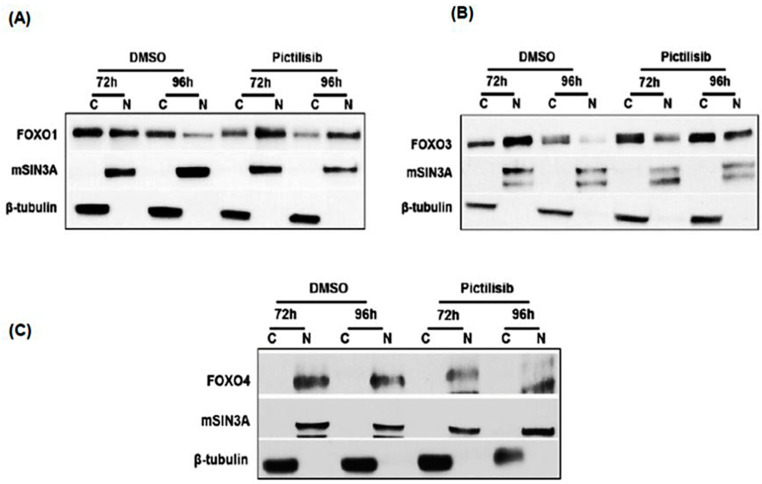
Enrichment of FOXO proteins in nuclear fractions after PI3Ki treatment. FOXO1 (**A**), FOXO3 (**B**), and FOXO4 (**C**) proteins from nuclear and cytoplasmic fractions of the vehicle or PI3Ki-treated (for 72 or 96 h) LS17T cells were resolved, blotted, and probed with respective antibodies: FOXO1, FOXO3, FOXO4, mSIN3A, and β-tubulin. Nuclear FOXOs were normalized to mSIN3A, and cytoplasmic FOXOs were normalized to β-tubulin. Notably, the nuclear fraction of FOXO1 at 72–96 h was elevated in the PI3Ki-treated cells (**A**), while FOXO4 proteins were restricted only to the nucleus with or without PI3K inhibition (1C). C = cytoplasmic; N = nuclear; vehicle = DMSO control.

Western blot was performed to characterize the translocation of FOXO1, FOXO3, and FOXO4 protein localization in MCA cells. The majority of the FOXO proteins were nuclear-localized following PI3K inhibition in LS174T cells (Figure 2). In DMSO-treated control cells, the nuclear fraction of FOXOs was decreased (Figure 2A,B). Surprisingly, FOXO4 proteins were totally restricted to the nucleus after 72 or 96 h of pictilisib and DMSO vehicle treatment (Figure 2C). Overall, of the FOXO family proteins, FOXO1 and FOXO3 were found to migrate into the nucleus as a result of PI3Ki treatment. Therefore, these results suggest that the nuclear fraction of FOXO1 proteins was comparable in PI3Ki-treated cells and are available for transcriptional enhancement of RTK activity and cell resistance associated with PI3K inhibition by pictilisib.

### 2.3. FOXO Knockdown in MCA Cells Mitigates PI3K and MAP Kinase Signaling Induced by Pictilisib Treatment

**Figure 3 ijms-24-12331-f003:**
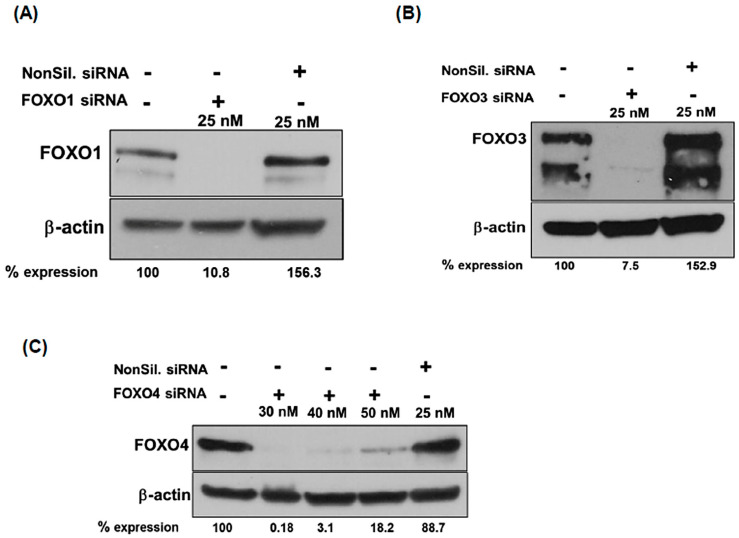
FOXO1, FOXO3, and FOXO4 were deleted in MCA cells by siRNA-mediated knockdown. (**A**–**C**): LS174T cells were transfected with siRNA smart pools (Dharmacon), targeting FOXO1, FOXO3, FOXO4, and non-silencing RNA as off-target transfection control. Western blot analysis showed effective knockdown of FOXO1, 3, and 4 in LS17T cells. Expression levels of proteins on Western blots were quantified by densitometry, and the percentages of FOXO protein expression relative to untreated (−) controls are shown below the blots. All values were normalized to β-actin as a loading control.

To understand the functional role of the FOXO protein in PI3K-inhibited MCA cells, we performed targeted gene knockdown in LS174T cells. FOXO siRNA (short-interference RNA) or non-silencing siRNAs were transfected into LS174T cells, and FOXO protein expression was assessed by Western blot analysis. Results indicate that FOXO protein was remarkably attenuated in LS174T cells following siRNA-mediated knockdown (Figure 3). Interestingly, FOXO1 and FOXO3 siRNAs efficiently knocked down target genes at lower concentrations compared to the FOXO4 siRNA gene attenuation that was achieved at higher siRNA concentrations (Figure 3C). Specificity was shown with the non-silencing siRNA having no effect on FOXO expression levels. Overall, FOXO target genes were efficiently knocked down using FOXO siRNAs, revealing the relevance of investigating FOXO factors in PI3K drug-induced resistance in MCA cells.

**Figure 4 ijms-24-12331-f004:**
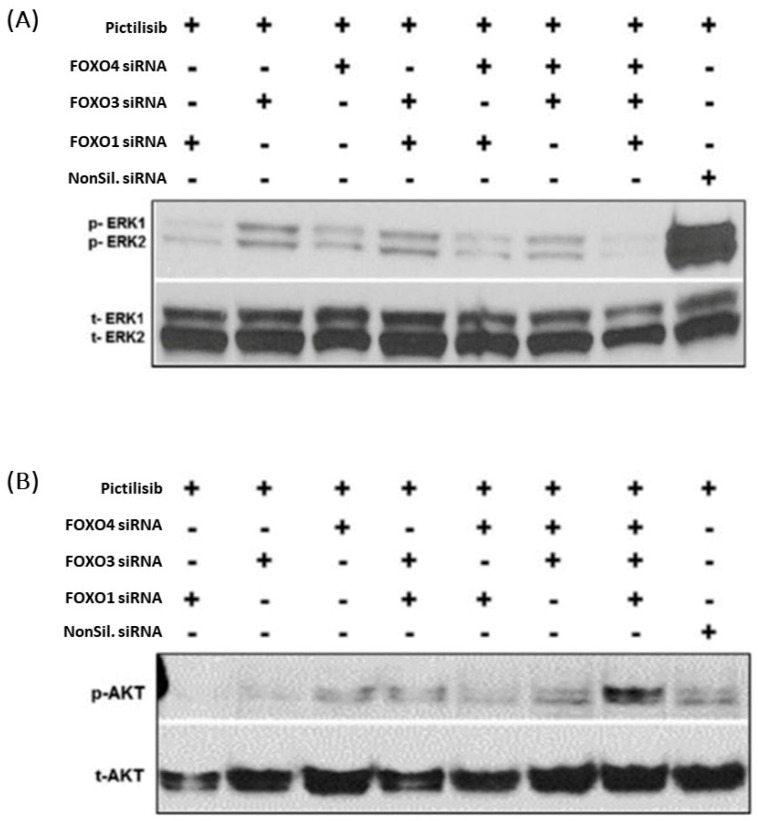
Rebound activation of pERK1/2 and pAKT seen in response to PI3K inhibition and FOXO knockdown. LS174T cells were transfected with FOXO1, FOXO3, or FOXO4 siRNA alone or in combination, grown for 24 h, and treated with PI3Ki for 96 h. (**A**) Western blots were performed using anti-pERK (pERK1 and ERK2), and (**B**) anti-pAKT and anti-total-ERK (tERK1 and ERK2), and total-AKT antibodies. The blot signals were quantified using densitometry and were normalized to non-silencing controls. Phospho-ERK1/2 levels were reduced in all PI3Ki-treated cells with FOXO knockdown compared to non-silencing controls (**A**). A more pronounced reduction in FOXO1 (**A**) and FOXO4 (**A**) siRNA-treated cells was seen than in FOXO3 siRNA-treated cells (**A**). Phospho-ERK1/2 levels were reduced when all three FOXO genes were knocked down, but this reduction was comparable to FOXO1 single knockdown, suggesting that FOXO1 may be the most critical FOXO protein for mediating rebound activation of pERK levels in response to PI3Ki-treatment. Similarly, phospho-AKT levels were reduced in FOXO1 (**B**), FOXO3 (**B**), and FOXO1 + FOXO4 siRNA-treated PI3Ki-resistant cells compared to FOXO3, non-silencing siRNA treated cells. Phospho-AKT levels were reduced with FOXO1 gene knockdown (**B**). Compared to all other FOXOs alone or together, knockdown indicates that the FOXO1 protein could be a critical modulator to influence PI3K inhibitor-treated resistant cell survival through AKT phosphorylation.

To determine the significance of FOXO protein attenuation during PI3K inhibition and resistance in MCA cells, we next examined the effect of FOXO knockdown in pictilisib-treated LS174T cells. The activity of downstream effector molecules (PI3K and MAP kinase signaling pathways) was evaluated in response to FOXO protein knockdown. In previous studies, we showed that ERK and AKT phosphorylation is initially reduced after short-term (24–48 h) pictilisib treatment but later increased after longer treatment periods (72–96 h) [13]. Here, FOXO1 siRNA transfection followed by pictilisib treatment for 96 h resulted in reduced pERK1/2 and pAKT activity compared to non-silencing or other FOXO siRNAs alone or in combination (Figure 4A,B). In contrast, phosphorylated-ERK activity remained elevated in non-silencing siRNA and pictilisib-treated MCA cells. Moreover, phosphorylation of AKT, a downstream factor of the PI3K survival pathway, was altered by FOXO siRNA in PI3Ki-induced LS174T-resistant cells (Figure 4B). FOXO1, FOXO3 alone, and FOXO4 siRNA showed markedly reduced AKT phosphorylation in PI3Ki-resistant LS174T cells (Figure 4B). FOXO1 alone knockdown resulted in the complete inhibition of pAKT, whereas other forms of FOXO siRNAs, including non-target siRNA, alone or in combination, led to less of a reduction in AKT phosphorylation in the PI3Ki-resistant cells. Thus, the resistance of MCA cells to pictilisib single-agent treatment was mitigated by FOXO1 functional gene attenuation and could be correlated to rebound activation of MAPK and AKT survival pathways. Thus, pictilisib single-agent induction of survival and growth in resistant cells depends on FOXO1-dependent pERK and pAKT activation.

### 2.4. FOXO1 Mediates the Redirection of Receptor Tyrosine Kinase mRNA Expression in PI3Ki-Induced LS174T-Resistant Cells

**Figure 5 ijms-24-12331-f005:**
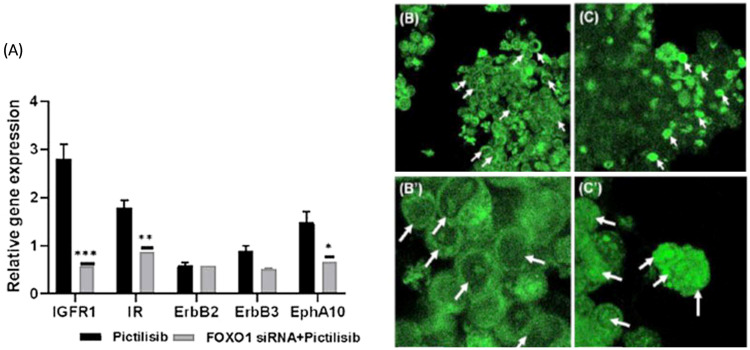
FOXO1-mediated differential expression of receptor tyrosine kinases (RTKs) in PI3Ki-induced resistant MCA cells. (**A**). Quantitative RT-PCR results show a reduction in *IGFR1*, *IR*, *ErbB2*, *ErbB3*, and *EphA10* mRNA relative gene expression levels in LS174T cells transfected with FOXO1 siRNA subjected to PI3Ki treatment. *ErbB2* and *ErbB3* mRNA relative gene expression was not significantly reduced. Error bars indicate the standard error of the mean of relative gene expression levels (n = 4). *p*-value significance (* *p* < 0.01, ** *p* < 0.001, and *** *p* < 0.0001). Error bars: standard error mean values were plotted. (**B**–**C’**): FOXO transcriptional activity monitored in PI3Ki-treated LS174T cells and DMSO vehicle control cells. (**B**,**B’**): FOXO1 transcriptional promoter element contained GFP reporter construct transfected 24 h, upon DMSO vehicle treatment for 96 h. Cytoplasmic GFP expression (white arrows) was seen in LS174T control cells. (**C**,**C’**): FOXO1 responsive promoter element contained GFP reporter construct transfected 24 h followed by PI3Ki treatment. After 96 h, nuclear GFP expression (white arrows) was seen in PI3Ki-resistant LS174T cells. Scale bar: (**B**–**C’**) 20 μm.

The contribution of RTK mRNA expression in MCA cell resistance was evaluated by RT-PCR to determine the possible correlation between pictilisib drug-induced resistance and FOXO1 attenuation in LS174T cells. The RTKs evaluated included the insulin receptor (*IR*), insulin growth factor receptor 1 (*IGF-R1*), *ErbB2*, *ErbB3*, and ephrin receptor A10 (*EphA10*). The results indicate that the rebound activity of FOXO1-mediated RTKs is seen with PI3K inhibition that is associated with pictilisib treatment of MCA cells (Figure 5). The statistical analysis revealed that remarkedly elevated levels of RTK mRNA expression were seen with PI3K inhibition at 96 h. In contrast, FOXO1 knockdown rescued pictilisib-induced rebound activity of RTK mRNA overexpression in the LS174T cells (Figure 5A). Further, we confirmed that RTK mRNA expression positively correlated with FOXO transcription factors using the Cignal Reporter GFP (green fluorescent protein) expression assay. As shown in Figure 5B, DMSO-treated MCA cells displayed cytoplasmic GFP expression. However, functional FOXO/TRE/GFP plasmid transfection followed by pictilisib treatment resulted in reporter GFP expression that was localized in the nucleus of LS174T cells (Figure 5C). This indicates that the nuclear fraction of FOXO1 promoter activity is required for the rebound activation of RTKs in pictilisib-treated MCA cells. Taken together, these data suggest that FOXO1-dependent RTK mRNA expression is elevated during pictilisib single-agent treatment.

### 2.5. FOXO1 Knockdown Sensitizes the Rebound Activity of RTKs during PI3K Inhibition in Drug-Induced Resistant MCA Cells

**Figure 6 ijms-24-12331-f006:**
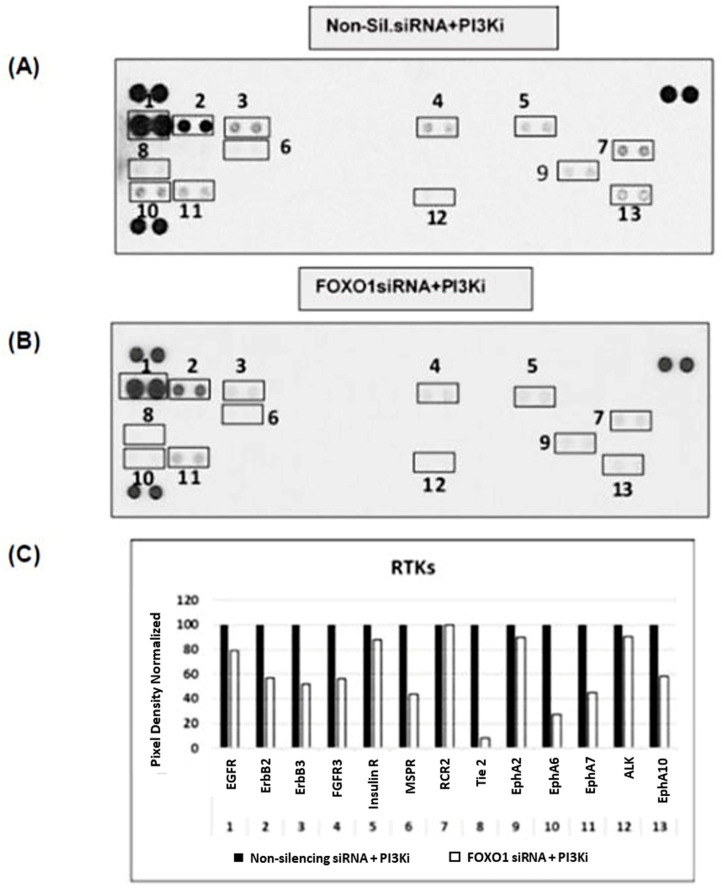
RTK phosphorylation in response to FOXO1 knockdown and prolonged exposure to PI3K inhibitor. (**A**,**B**): After 96 h, FOXO1 and non-silencing siRNA followed by PI3Ki treated LS174T cell lysates were incubated with RTK profiler antibody array containing 49 RTKs (R&D Biosystems), followed by the anti-phospho-tyrosine antibody. The RTKs that were elevated and downregulated in the boxes indicate RTK expression. (**C**): Pixel densities were quantified using ImageJ analysis software 1.44. The numbers on the *x*-axis correspond to RTKs and fold elevation relative to non-silencing siRNA + PI3Ki controls. The *x*-axis number 1 to 13 corresponds to EGFR, ErbB2, ErbB3, FGFR3, Insulin R, MSPR, RCR2, Tie 2, Eph A2, Eph A7, ALK, and Eph A10. These RTKs activity was normalized with non-silencing siRNA + PI3Ki.

The potential of FOXO1 as a regulator of the PI3K pathway led us to investigate the loss of the FOXO1 functional gene during pictilisib-induced rebound activity of RTKs and upstream elements of these signaling pathways. The RTK proteome profile array was analyzed in cells transfected with FOXO1 and non-silencing siRNA along with pictilisib treatment. Results indicate that FOXO siRNA followed by pictilisib treatment exhibited less phosphorylation of IGF-R1, IR, EphA10, EphA7, ERbB2, and ErbB3 at 96 h, whereas transfection with non-silencing siRNA led to significantly elevated RTK phosphorylation (Figure 6). Our previous reports demonstrated that pictilisib has a synergistic effect. For instance, linsitinib (IR and IGFR1 inhibitor) vs. pictilisib and lapatinib and EGFR and ErbB2 inhibitor vs. pictilisib. This combinational synergism resulted in a loss of viability in MCA cell lines [13]. However, ephrin receptors have been identified as key modulators to regulate RAS/MAPK and PI3K/AKT pathways in tumor progression [25]. At present, there are no inhibitors available to target EphA7 or EphA10. Taken together, the results show that FOXOs, especially FOXO1, are sensitizing pictilisib drug-induced rebound activity by attenuating RTKs in MCA cells.

### 2.6. PI3Ki and FOXO1i Synergy in MCA Cell Lines

A major pathway targeted by therapeutic drugs in human cancers is the PI3K/AKT/mTOR signaling pathway. Several small-molecule inhibitors have been developed to target PI3K; however, the development of PI3K-dependent resistance mechanisms has limited their success in the clinical spectrum [1]. In our previous studies, we evaluated IC50 values and initial sensitivity to pictilisib [13]. However, resistance occurred within 72 h of pictilisib treatment. Here, we investigated the hypothesis that combined inhibition of PI3K and FOXO1 would diminish drug-induced resistance. To test this hypothesis, MCA cell lines (LS174T and RW7213) were treated with increasing concentrations of pictilisib and the FOXO1 inhibitor (AS1842856). Our results demonstrate that both MCA cell lines are sensitized after individual treatments for 96 h (Supplementary Figure S1A,B). A four-parameter logistic/sigmoidal dose–response model was used to assess the sensitivity of both MCA lines to FOXO1i (IC50: 0.28 µM (LS174T) and 0.17 µM (RW7213)). Subsequently, synergistic responsive experiments were performed with AS1842856 and pictilisib in the MCA cells. Importantly, the combinational treatment of pictilisib and AS1842856 synergistically increased cell cytotoxicity in both MCA cell lines (Supplementary Figure S1C,D). A combination index (CI) value of <1 [26] was seen in both MCA cell lines (CI = 0.54 for LS174T and CI = 0.47 for RW7213), suggesting synergy between the two drugs in reducing MCA viability in vitro. Thus, these results demonstrate that the drug-induced resistance to pictilisib single-agent inhibition in MCA cells could be bypassed using FOXO1i and PI3Ki combination therapy.

## 3. Discussion

Although the PI3K/AKT/mTOR axis is a target for cancers, the current use of small molecule inhibitors against this axis is limited by the development of drug resistance in patients [23,27,28]. We confirmed that similar resistance occurs with MCA cells in vitro and that pictilisib-induced drug-resistance can lead to cancer cell survival and aggressiveness in MCA cells. Additionally, studies have reported that FOXO proteins are putative markers for poor chemotherapeutic response in various cancer models [24,29]. However, the molecular mechanism of drug-induced phase resistance in MCA is still uncertain.

In the present study, we explored the possible underlying mechanism of FOXO protein involvement in MCA cell drug-induced resistance that is mediated by pictilisib administered as a single agent. The results revealed that pictilisib treatment led to an increased accumulation of nuclear FOXO1 compared to vehicle-treated LS174T cells. FOXO transcription factors are highly competitive transcriptional binders involved in determining the fate of target cells [30]. Nuclear FOXO1 is most likely to be involved in cell fate decisions with the influence of RTK gene transcription in resistant cells. MCA cells are sensitized during pictilisib treatment by FOXO1 proteins migrating from the cytoplasm to the nucleus and their involvement in the process of drug-induced resistance. It could be possible that acute phase resistance to pictilisib leads to FOXO1-dependent RTK gene transcription and reduction in pro-apoptotic gene (e.g., BIM) activity that results in MCA cell survival following the drug treatment. For instance, a recent study showed that FOXO1 influences AKT phosphorylation in hepatocytes by suppressing the expression of Tribble 3 (Trb3), a pseudokinase capable of binding to AKT to block phosphorylation [31]. However, the rebound activation of RTK receptors, the phosphorylation of ERK and AKT, and the nuclear accumulation of FOXO1 were clearly observed in response to pictilisib treatment at 96 h in MCA cells. It appears there is clear evidence that the FOXO1-dependent positive feedback loop to the MAPK and PI3K/AKT pathways could support the development of drug-induced resistance and survival in MCA cells. Indeed, several studies have reported that inhibition of the PI3K/AKT axis leads to enhanced phosphorylation of ERK and AKT in several cancer models, including breast, prostate, and lung cancers [32,33,34]. However, Serra et al. reported that acquired ERK activity is a major cause of resistance to PI3K inhibitors in cancer treatment [34]. Similarly, our results show that pictilisib-treated LS174T cells have elevated ERK1/2 phosphorylation. However, FOXO siRNA followed by pictilisib treatment of LS174T cells leads to a reduction in IR, IGF-R1, ErbB2, ErbB3, EphA7, and EphA10 receptor activity and phosphorylation of AKT and ERK. These results suggest that FOXO-based promoter activity is essential to synthesize target gene receptors which are involved in cell survival and growth in resistant cancer cells [23,27,31,35]. In addition, earlier studies demonstrated that the inhibition of the PI3K/AKT axis inhibition leads to the nuclear localization of FOXO proteins and promotes transcriptional activation of RTK receptors through ERK signaling in breast cancer cells [33,34]. Here, we observed that targeting FOXO proteins by blocking functional activity with siRNAs resulted in decreased AKT and ERK phosphorylation in MCA cells. Thus, the FOXO1-mediated attenuation of RTKs (MAPK and PI3K signaling pathways) represents a more proximal mechanism that controls pictilisib drug-induced phosphorylation of ERK (Supplementary Figure S2). We suggest that FOXO1 acts as a pleiotropic effector on multiple signaling pathways, targeting drug-induced resistance by interfering with RTK signaling and the apoptotic pathway. In addition, the utilization of a small-molecule inhibitor against FOXO1 could effectively target PI3Ki-induced resistance and reduce ERK and AKT activity.

FOXO1i (AS1842856) is emerging as a selective chemotherapeutic drug against FOXO1 which is currently used for the treatment of Type II diabetic mullites (T2DM) [36,37]. Despite the recognition of FOXO1 nuclear localization in the development of pictilisib drug-induced resistance, how effectively FOXO1i treatment with a combination of PI3Ki causes ERK activation remains elusive. Based on previous studies, inhibition of the PI3K/AKT axis induces nuclear accumulation of FOXO proteins [33,34,38,39]. Our results suggest that pictilisib single-agent treatment increases RTK expression and that ERK1/2 phosphorylation is mediated by the nuclear localization of FOXO1. Importantly, we show that this effect is completely abolished by FOXO siRNA-mediated knockdown or FOXO1 inhibitor administered as a co-treatment with pictilisib. Our findings propose that pictilisib single-agent treatment induces nuclear localization of FOXO1 transcriptional activity, which is rescued by FOXO1 siRNA or the combinational inhibition of pictilisib with a FOXO1 inhibitor. FOXO1i (AS1842856) and pictilisib synergy represent a key mechanism responsible for RTK rebound activity and drug resistance in MCA cells. Therefore, this study suggests that FOXO1 involvement is a critical event in PI3K/AKT axis inhibition that mediates resistance in MCA cells. Thus, our results suggest that (a) drug-induced resistance by pictilisib is mediated by FOXO1 transcription factors in MCA cells; (b) FOXO siRNA attenuates RTK rebound activity and diminishes ERK and AKT phosphorylation; (c) FOXO1i and PI3Ki combinational synergy are more effective in reducing MCA cell viability and increasing apoptosis.

In summary, we demonstrated that FOXO1 nuclear accumulation correlates with the acute resistance created in MCA cells following pictilisib treatment. We provide evidence that FOXO1 regulates RTK rebound activity in pictilisib drug-induced resistant cells. Further, FOXO siRNA attenuates phospho-ERK1/2 and phospho-AKT levels in pictilisib given as a single agent to MCA cells. Most importantly, FOXO1i (AS1842856) and pictilisib combinational synergy increase efficacy through cell toxicity and apoptosis. Although these data were generated from in vitro studies, it is likely that in vivo analyses will confirm the findings and the potential therapeutic value of FOXO1i/PI3Ki treatment in MCA cells. We speculate that FOXO1 might be a co-target to rescue PI3Ki single-agent resistance in MCA therapy.

## 4. Materials and Methods

### 4.1. Cell Lines

Mucinous colorectal adenocarcinoma cell lines (LS174T and RW7213) were cultured as described in our previous studies [13]. Briefly, LS174T (American Type Culture Collection; Manassas, VA, USA) and RW7213 (gifted from John Mariadason; Ludwig Cancer Institute, Melbourne, Australia) cell lines were grown as monolayer cultures in RPMI1640 (Hyclone Laboratories, Logan, UT, USA) supplemented with 4.5 g/L glucose (Invitrogen, Carlsbad, CA, USA), 10% FBS (Invitrogen, Carlsbad, CA, USA), 2 mM L-glutamine, 20 mM HEPES, 1X MEAA, 100 IU/mL penicillin, and streptomycin in a humidified atmosphere of 5% CO_2_ at 37 °C. The cell lines were tested for interspecies cross-contamination and mycoplasma infection and authenticated by short tandem repeat [10] analysis using 16 STR markers (IDEXX Bio-Analytics, Columbia, MO, USA).

### 4.2. Immunocytochemistry

LS174T or RW7213 cells were grown on chamber slides and treated for 72 and 96 h with pictilisib or Dimethyl sulfoxide (DMSO) vehicle control. Subsequently, the cells were washed with 1X cold PBS and fixed in 4% PFA. Following incubation with blocking solution (0.1% TritonX-100 at RT for 1 h), the cells were incubated with rabbit-anti FOXO1 (1:50 dilution; Cell Signaling Technology, Boston, MA, USA) in a humid chamber overnight at 4 °C. For the visualization of FOXO1, Alexa594 conjugated anti-rabbit secondary antibody (Invitrogen, Carlsbad, CA, USA) was applied to the cells for 1 hour at RT and DAPI-containing anti-fade mounting media was used for nuclear counterstaining. The positive cytoplasmic and nuclear-stained cells were counted in both PI3Ki and DMSO vehicle control cells.

### 4.3. Fluorescence Microscopy

Images of pictilisib and DMSO-treated LS174T and RW7213 cells were obtained using multiphoton confocal microscopy (Leica TCS SP8 MP Microsystems Inc., Buffalo Grove, IL, USA). Images were captured using AxioVision software version 4.8 and quantified using meta soft analysis.

### 4.4. FOXO Signal Reporter Assays

LS174T cells were transfected using a Cignal FOXO reporter kit (cat # CCS-6022G Qiagen, Germantown, MD, USA) according to the manufacturer’s protocol. In brief, LS174T cells were transfected with FOXO/TRE/GFP construct for 24 h, followed by PI3Ki treatment for 96 h. GFP fluorescence identification of transfected cells was performed using ImageXpress Ultra microscope and MetaXpress software version 2.6.

### 4.5. siRNA Knockdown Studies

Three pairs of siRNA Smart pool reagents (cat # M-003006-03-0010; cat # M-003007-02-0010; cat # M-003016-02-0010 Dharmacon, Lafayette, CO, USA) were used against FOXO1, FOXO3, and FOXO4 gene targets. The siRNA transfection protocol was performed according to the manufacturer’s instructions with FOXO siRNAs introduced with DharmaFECT transfecting reagent (cat # 8T-2001-01). As a transfection control, on-target and off-targeting siRNA pools were used (cat # D-001810-10-20). Seventy-two hours after transfection, 12.5–30 nM siRNA-treated cell proteins were harvested, and total protein concentration was measured using the BCA method.

### 4.6. Western Blots

Total cell proteins were lysed in radioimmunoprecipitation (RIPA) lysis buffer containing protease phosphatase cocktail inhibitor (Cell Signaling; Danvers, MA, USA). After centrifugation (2000× *g* at 4 °C), the supernatant was mixed with an equal volume of Laemmli sample buffer and boiled. The denatured proteins were resolved by precast SDS-PAGE (sodium dodecyl sulphate–polyacrylamide gel electrophoresis) 4–15% gradient gels followed by electrotransfer to polyvinylidene fluoride or polyvinylidene difluoride (PVDF) membranes (Millipore; St. Louis, MO, USA). The blots were blocked with 5% skim milk for 1 h at RT and probed with the following antibodies overnight at 4 °C: anti-FOXO1 (cat # 8H0003845), anti-FOXO3 (cat # 550485), FOXO4 (cat # 550982), phosphorylated ERK (pERK) (cat # 4376), total ERK (tERK) (cat # 4695), phosphorylated AKT (pAKT) (cat # 4060), and total AKT (tAKT) (cat # 4691), all from Cell Signaling, and anti-β-actin (cat # A5441, Sigma Aldrich; Saint Louis, MO, USA). The blots were then washed three times with 1X TBST (Tris-buffered saline with 0.1% Tween^®^ 20) detergent and incubated with the appropriate secondary antibody for 1 h at RT. The antigen–antibody complex was detected by enhanced chemiluminescence according to the manufacturer’s instructions (ThermoFisher; Denver, CO, USA). Signals on the blots were quantified by densitometry software (Quantity one 4.6.5, Bio-Rad, Hercules, CA, USA). All the Western blots were performed in triplicate.

### 4.7. RTK Arrays

The RTK (Receptor Tyrosine Kinase) Proteome Profile Array Kit (cat # 8ARY001B, R&D systems, Minneapolis, MN, USA) was used to measure alterations in RTK phosphorylation in response to FOXO1 siRNA, non-silencing siRNA, or following PI3Ki treatment in LS174T cells as previously described [13]. Following treatments, the cells were washed with cold PBS (phosphate-buffered saline), lysed in NP40 lysis buffer, and 300 μg of cell lysates were incubated with blocked membranes overnight. Membranes were subsequently washed and incubated with an HRP (horseradish peroxidase)-conjugated anti-phosphotyrosine antibody (supplied with the kit) for 1 h at RT, washed, and ultimately incubated with a chemiluminescent substrate according to the manufacturer’s protocol. The membrane was then exposed to X-ray film, and the results (image spot intensity and pixel density) were quantified by Image J analysis software 1.44.

### 4.8. Quantitative Real-Time PCR

Pictilisib-induced, FOXO-dependent RTK target gene expression in MCA cells was assessed by quantitative real-time PCR (qRT-PCR). Total RNA was extracted from FOXO1 and non-silencing siRNA expressing pictilisib (PI3Ki)-treated LS174T cells using the ToTally RNA kit (cat # AM1910, Ambion, Austin, TX, USA). RNA was treated with RNase-free DNase (Invitrogen) for 30 min at RT, and the first-strand cDNA was synthesized from 1 μg of DNase-treated RNA by MMLV reverse transcriptase (cat # New England Biolabs, Ipswich, MA). For qRT-PCR, 1μL of cDNA was mixed with 10 μL of 2X Fast SYBR green mix (cat # Applied Biosystems, Foster, CA, USA), oligonucleotides, and RT-PCR run using ABI 7500 machine (Applied Biosystems). Supplementary Table S1 lists the oligonucleotides used for the amplification of IGF-1R, IR, ErbB2, ErbB3, EphA10, and GAPDH as a reference control. In order to rule out probable contamination of genomic DNA, negative controls were performed in parallel by directly using RNA as a template for PCR. For data analysis, target gene Ct (cycle threshold) values were normalized to the GAPDH housekeeping gene and 2ddct values were reported as fold change relative to the control.

### 4.9. Cell Viability Assays

Cells were treated for 96 h with pictilisib (PI3Ki) (16 nM to 1 mM) or AS1842856 (FOXO1i) (1 nM to 1 mM), and cell viability was measured using the WST-1 cell viability assay (cat # 2198 Bio-Vision; Milpitas, CA, USA). The concentration of the drug resulting in 50% of maximal inhibition (IC50) was calculated from a four-parameter sigmoidal dose–response model (XLfit, IDBS). In vitro synergy studies were performed by the addition of PI3K and FOXO1 inhibitors in a fixed ratio ranging from 0.0039X to 4X IC50 of each drug, either alone or in combination. The synergy between drugs was followed based on our previously described studies [13] using the Compusyn program described by Chou and Talalay [26].

### 4.10. Statistical Analyses

Statistical analysis of all data was presented as mean ± SEM using Graph Pad Prism 8. A comparison between groups was carried out by a two-tail *t*-test, and values of *p* < 0.05 were considered statistically significant.

## References

## Data Availability

Once the database is finalized, it can be made available for data-sharing.

## Supplementary Materials

The supporting information can be downloaded at: https://www.mdpi.com/article/10.3390/ijms241512331/s1.

## Abbreviations

AKT	AKT kinase (Protein Kinase B or PKB)
AS1842856	FOXO1 inhibitor
BCA	Bicinchoninic Acid
BRAF	Murine sarcoma viral oncogene homolog B
DAPI	4,6-diamidino-2-phenylindole
DMSO	Dimethyl sulfoxide
EphA7	EPH Receptor A7
EphA10	EPH Receptor A10
ErbB2	Erb-B2 Receptor Tyrosine Kinase 2
ErbB3	Erb-B2 Receptor Tyrosine Kinase 3
FGFR 3	Fibroblast Growth Factor Receptor 3
GFP	Green Fluorescent Protein
IGF-R1	Insulin Growth Factor Receptor 1
INSR	Insulin Receptor pERK: phosphor-ERK1&2
KRAS	Kirsten rat sarcoma virus
MAP Kinase	Mitogen-activated protein kinase
MSP R	Macrophage Stimulating 1 Receptor
mTOR	Mechanistic Target Of Rapamycin Kinase
pAKT	Phosphor-AKT
PBS	Phosphate-buffered saline
PFA	Paraformaldehyde
PI3K	Phosphatidylinositol Kinase 3
pictilisib	PI3K inhibitor
ROR2	Receptor Tyrosine Kinase-Like Orphan Receptor 2
S6 Kinase	Ribosomal Protein S6 Kinase
siRNA	Small interfering RNA
Tie-2	Tyrosine Kinase with Immunoglobulin-Like and EGF-Like Domains 2
TRE	Tetracycline response element (TRE)
