# Supplementary material for: Pictilisib-Induced Resistance Is Mediated through FOXO1-Dependent Activation of Receptor Tyrosine Kinases in Mucinous Colorectal Adenocarcinoma Cells
Source: Int J Mol Sci. 2023 Aug 2;24(15):12331. doi: 10.3390/ijms241512331 (PMC10418489; doi:10.3390/ijms241512331)
Supplement: Supplementary file 1 [file ijms-24-12331-s001.zip › ijms-2521760-supplementary.pdf]

International Journal of *Molecular Sciences*

MDPI

Pictilisib-Induced Resistance Is Mediated through FOXO1-Dependent Activation of Receptor Tyrosine Kinases in Mucinous Colorectal Adenocarcinoma Cells

Murali R. Kuracha [1],*, Venkatesh Govindarajan [2], Brian W. Loggie [3], Martin Tobi [4] and Benita L. McVicker [1,5],*

1 Department of Internal Medicine, University of Nebraska Medicine, Omaha, NE 68198, USA

2 Department of Medical Education, Creighton University School of Medicine, Omaha, NE 68178, USA

3 Department of Surgery, Creighton University School of Medicine, Omaha, NE 68124, USA

4 Research and Development Service, Detroit VAMC, Detroit, MI 48201, USA

5 Research Service, Nebraska-Western Iowa Health Care System, Omaha, NE 68105, USA

* Correspondence: murali.kuracha@siriusmindshare.com (M.R.K.); bmcvicker@unmc.edu (B.L.M.); Tel.: +1-402-995-3369 (B.L.M.)

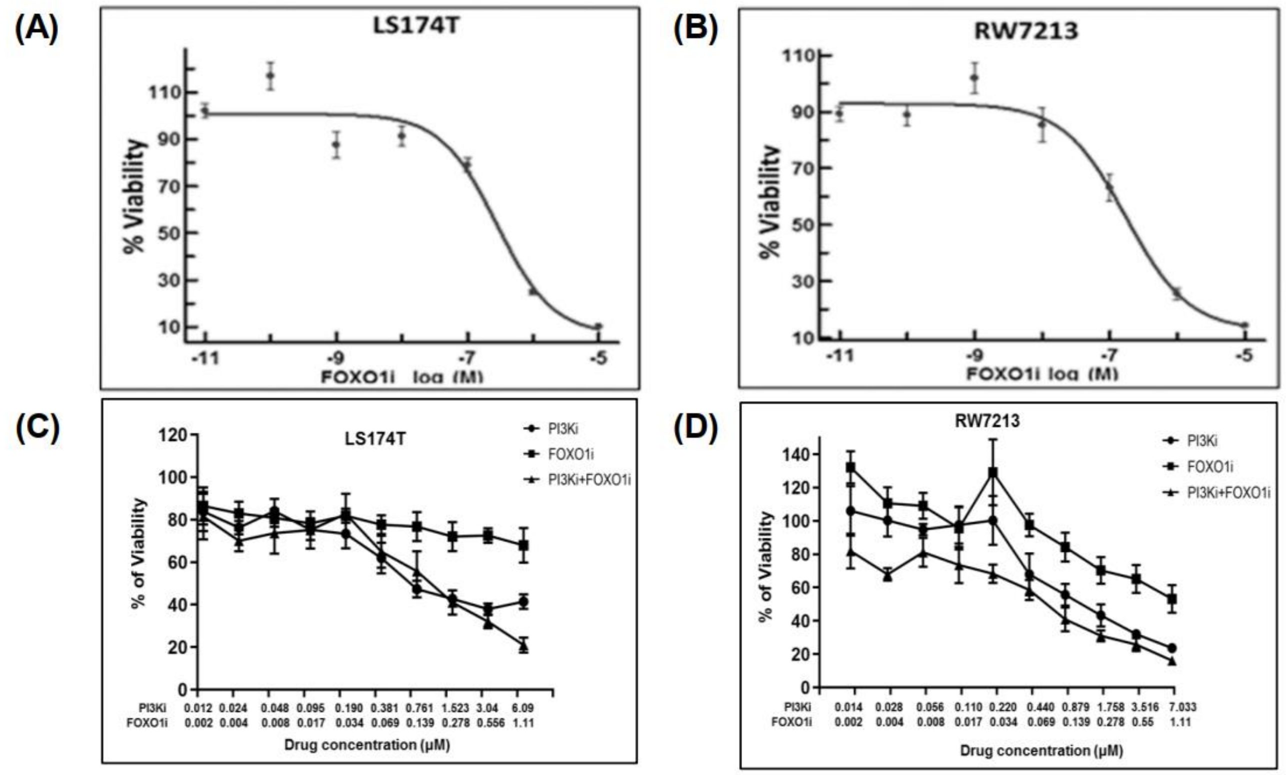

Figure S1. MCA cell lines are sensitive to FOXO1 inhibition (FOXO1i). **A-B:** LS174T and RW7213 cells were treated with the FOXO1 inhibitor (AS1842856), for 96 hours (n=6 for each data point). A four-parameter logistic/sigmoidal dose-response model was used for curve fitting. Both MCA lines were sensitive to FOXO1i (IC50: 0.28 μM (LS174T) and 0.17 μM (RW7213)). Error bars represent the standard error of the mean (n=6). Y-axis: % viability normalized to vehicle-treated controls. **C-D:** Synergistic responsive experiments were performed with FOXO1i and PI3Ki, in LS174T and RW7213 cells. Combinational synergy (CI<1) was seen in both LS174T and RW7213 cells. LS174T and RW7213 lines were treated with FOXO1i alone (■), PI3Ki alone (●), or a combination of the two inhibitors in a fixed ratio (▲) for 96 hours (n=6 for each data point). Error bars are standard deviations. X-axis: concentration of inhibitors in μM. Y-axis: % viability normalized to vehicle-treated controls.

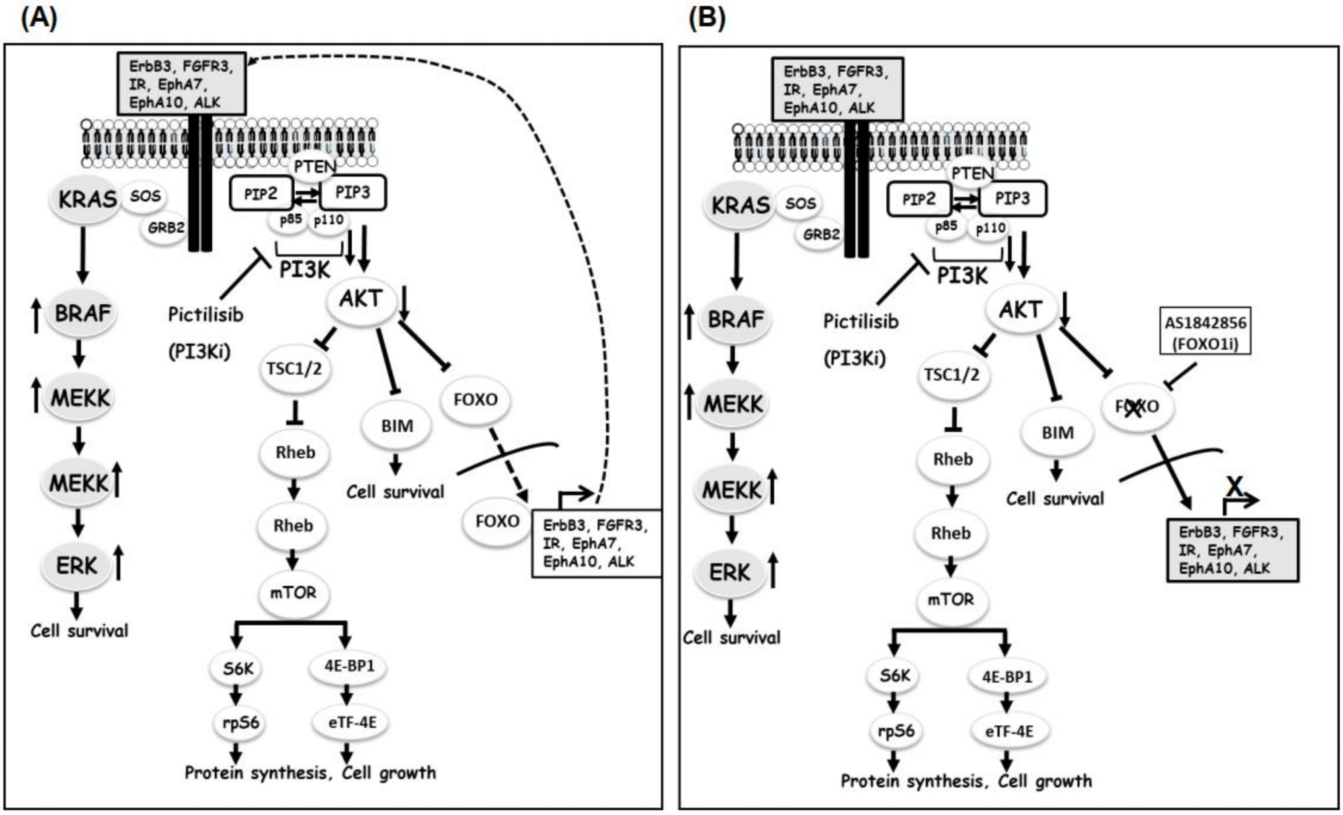

Figure S2. Pictilisib-induced feedback inhibition rescued by cotargeting with FOXO1 dependent signaling pathways. **A.** Rebound activation of FOXO dependent RTKs signaling pathways to pictilisib induced acute resistance in Mucinous Colorectal Adenocarcinoma cells (LS174T, RW7213). **B.** Combinational therapy, PI3Ki co-targeted with FOXO1i inhibits FOXO-mediated activation of RTK transcription.

Table S1. FOXO1 dependent RTKs target gene amplifying primers list

S.No.	Gene	Primer Sequence	Orientation
1	IGF-1R	5′CTCCTGTTTCTCTCCGCCG 3′	Forward
2	IGF-1R	5′ATAGTCGTTGCGGATGTGCGAT 3′	Reverse
3	IR	5′CTTCAGTTCGTGTGTGTGGAGACAG 3′	Forward
4	IR	5′CGCCCTCCGACTGCTG 3′	Reverse
5	ErbB2	5′AGCCTTGCCCCATCAACTG 3′	Forward
6	ErbB2	5′AATGCCAACCACCGCAGA 3′	Reverse
7	ErbB3	5′CCCTGCCATGAGAACTGCAC 3′	Forward
8	ErbB3	5′TCACTGTCAAAGCCATTGTCAGAT 3′	Reverse
9	EphA10	5′GTTCAGCCAAAGAGATGCCTAGGCTCAC 3′	Forward
10	EphA10	GTTCAGCCAAAGAGATGCCTAGGCTCAC 3′	Reverse
11	GAPDH	5′AATGAAGGGGTCATTGATGG 3′	Forward
12	GAPDH	5′AAGGTGAAGGTCGGAGTCAA 3′	Reverse
